# Spinal Cord Infarction After Transarterial Chemoembolization for Hepatocellular Carcinoma: A Case Report

**DOI:** 10.7759/cureus.85147

**Published:** 2025-05-31

**Authors:** Tho Tran Dinh, Sy Than Van, Vinh Chu Van, Phuc Chu Minh

**Affiliations:** 1 Department of Hepatobiliary Surgery, Viet Duc University Hospital, Hanoi, VNM; 2 Department of Radiology, Viet Duc University Hospital, Hanoi, VNM; 3 Department of Neurology, Viet Duc University Hospital, Hanoi, VNM

**Keywords:** corticosteroid, hepatocellular carcinoma, ischemia, spinal cord infarction, transarterial chemoembolization

## Abstract

Spinal cord infarction is a rare but serious complication following transarterial chemoembolization (TACE) for hepatocellular carcinoma (HCC). We report a case of a 33-year-old male with a history of chronic hepatitis B and multiple prior TACE sessions who developed acute paraplegia after undergoing another TACE procedure. With immediate administration of high-dose corticosteroids and supportive care, the patient had partial neurological recovery. This case highlights the importance of recognizing extrahepatic arterial supply and the potential neurological risks associated with TACE.

## Introduction

Hepatocellular carcinoma (HCC) is one of the most common cancers in Asia, with transarterial chemoembolization (TACE) being a well-established treatment option, particularly for patients with unresectable tumors [1,2]. Although generally safe, TACE can result in various complications, including post-embolization syndrome, abscess, liver failure, and rare neurological complications such as spinal cord infarction. We report a rare case of spinal cord infarction following TACE in a young patient with HCC, and review the relevant literature about this uncommon complication. This case report has been reported in line with the Surgical Case Report (SCARE) Criteria [3].

## Case presentation

A 33-year-old male patient was referred from a provincial general practice hospital due to inadequate response to previous TACE procedures. He had a history of unmanaged chronic hepatitis B and underwent emergency transarterial embolization for ruptured HCC a year prior. Two additional TACEs were performed within the same year, but tumor control remained unsatisfactory.

**Figure 1:**
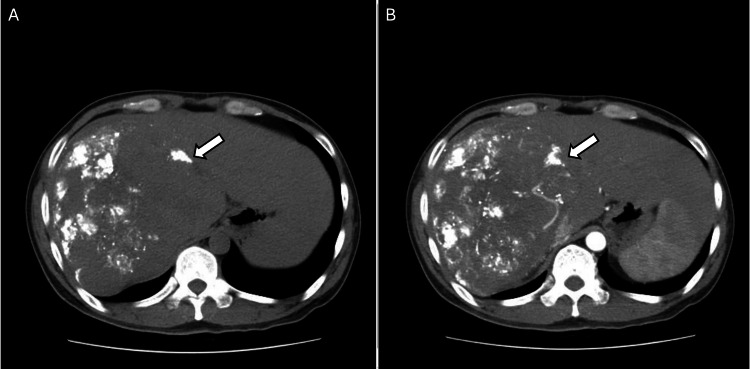
Contrast-enhanced computed tomography (CT) images obtained before embolization. A) Pre-contrast axial CT image showing the residual tumor (arrowhead) in the right hepatic lobe. (B) Post-contrast axial CT image demonstrating enhancement of the residual tumor (arrowhead), which is supplied by the right hepatic artery and the right inferior phrenic artery.

At admission, the patient was in good general condition without abdominal pain. Computed tomography (CT) revealed a large tumor measuring 13 × 12 cm occupying the entire right liver, along with a smaller lesion (4 × 3 cm) in segment II (Figure 1). Both lesions contained embolization material but still demonstrated partial arterial enhancement in the arterial phase and washout in the venous phase. Laboratory tests showed a positive hepatitis B surface antigen (HBsAg) and an elevated serum alpha-fetoprotein (AFP) level of 369,000 U/mL, while liver function tests and complete blood count were within normal limits. Given the tumor burden and inadequate prior response, repeat TACE was planned.

**Figure 2:**
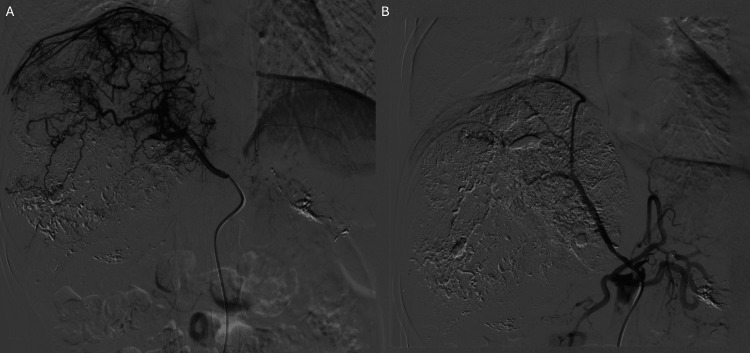
Digital subtraction angiography (DSA) images of the right inferior phrenic artery. (A) Selective angiography of the right inferior phrenic artery demonstrates hypervascularization of the tumor, with extensive tumor vascularity supplied by this artery. (B) Post-embolization angiography showing successful occlusion of most tumor-feeding branches.

During angiography, tumor supply was noted from both the right hepatic artery and the right inferior phrenic artery. Embolization was performed using a combination of lipiodol and drug-eluting beads (Figure 2). Approximately 15 minutes after the procedure, the patient reported a sudden onset of numbness in the lower extremities. Spinal cord ischemia was suspected, and an emergency neurology consultation was obtained. High-dose methylprednisolone was promptly administered, with an initial bolus of 30 mg/kg followed by a continuous infusion of 5.4 mg/kg/h over 23 hours. A neurosurgeon was consulted - there was no indication for decompression surgery.

**Figure 3:**
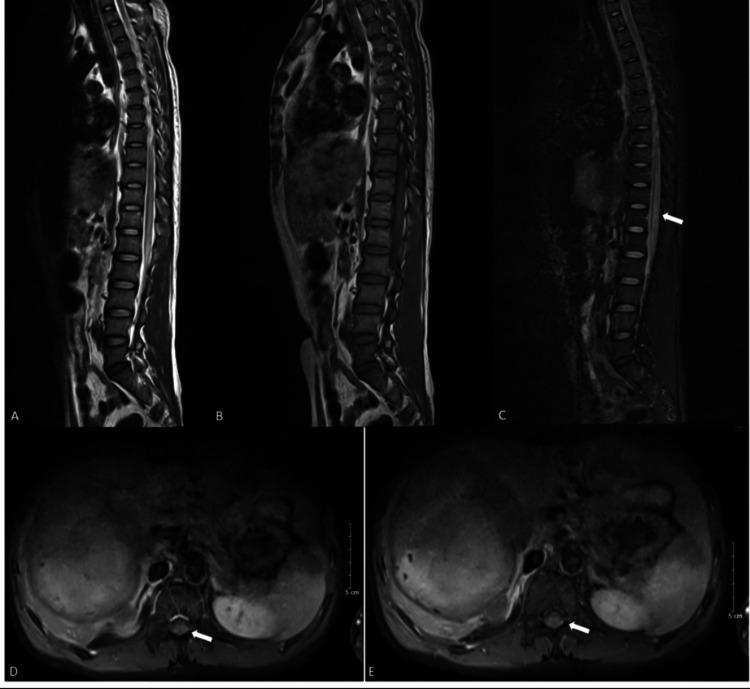
Magnetic resonance imaging (MRI) of the thoracic-lumbar spine on the next day after embolization. (A) T2-weighted image (T2W) showing hyperintense signal within the spinal cord at the T9-L1 level. (B) T1-weighted image (T1W) demonstrating hypointense signal in the same region. (C) Short tau inversion recovery (STIR) image revealing a hyperintense signal, indicating edema. (D, E) Post-contrast axial images of the corresponding spinal level showing persistent contrast enhancement of the affected region, suggesting ongoing vascularity or inflammation.

**Figure 4:**
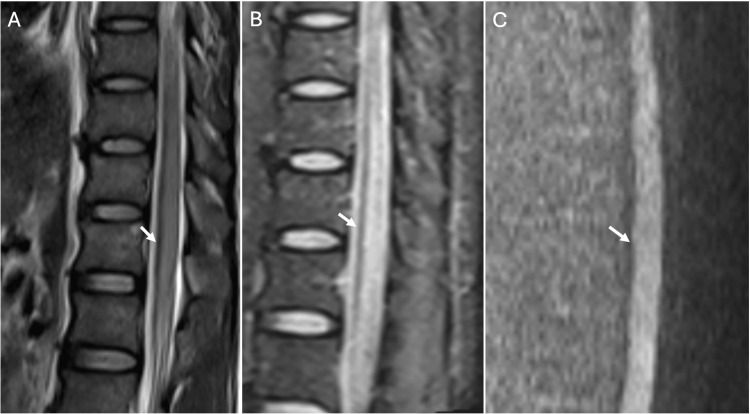
Magnetic resonance imaging (MRI) of the thoracic-lumbar spine, zooming in on the lesion at T9 level. (A) T2-weighted image (T2W) showing hyperintense signal within the spinal cord at the T9-L1 level. (B) Short tau inversion recovery (STIR) image revealing a hyperintense signal, indicating edema. (C) Diffusion-weighted image of the corresponding spinal level showing no restriction of the affected spinal cord, compatible with edema.

An hour post-procedure, the patient developed complete loss of sensation below the T9 level, with a Medical Research Council (MRC) motor score of 1/5 in the right leg and 2/5 in the left leg. Voluntary anal contraction was preserved. Magnetic resonance imaging (MRI) performed the following day confirmed ischemia at the T9 spinal cord level (Figures 3-4).

Over the next several days, the patient received maintenance corticosteroid therapy (2 mg/kg/day) and early rehabilitation, including pain management and physical therapy. Urinary retention was managed with a Foley catheter, followed by intermittent clamping on day 3 to facilitate bladder function recovery. The patient experienced transient constipation, which was alleviated with enemas. After a one-week hospital stay, he was discharged to a local rehabilitation center. At discharge, partial motor recovery was noted in the left leg (motor score 3/5), while sensory deficits and right leg weakness persisted. At follow-up through video consultation 8 months after the event, the patient has regained most of his sensory, bowel, and bladder function, and he is able to walk unassisted with a cane. His motor function score for the left leg was 4/5, and for the right leg was 3/5, as examined by a local general physician.

## Discussion

TACE is an effective and commonly used treatment for HCC, though it is associated with potential complications. Typical adverse effects include post-embolization syndrome (fever, nausea, and abdominal pain), elevated transaminases, and transient leukocytosis [4]. While neurological complications are rare, spinal cord infarction remains a serious and potentially debilitating event, occurring in approximately 0.3% of cases [5,6].

The development of spinal cord infarction following TACE is often attributed to unintentional embolization of the anterior spinal artery, or embolization of the artery of Adamkiewicz via extrahepatic collateral circulation. The right inferior phrenic artery, which commonly supplies large HCC tumors, has been implicated in such complications. Additionally, patients with prior TACEs, multifocal or infiltrative HCCs may develop altered vascular anatomy, increasing the risk of inadvertent embolization of intercostal or lumbar arteries. There might be small arterial branches from an inferior phrenic artery or intercostal arteries connecting with the anterior spinal arteries. These branches are usually very small, and they can be hard or even impossible to visualize on angiography [5-7]. Thus, for these patients, the risk of spinal infarction should be considered, and the radiologists must be very meticulous and vigilant. Neurological symptoms can appear immediately or within several hours post-TACE, ranging from mild paresthesia to complete paraplegia. Diagnosis is primarily confirmed through MRI, which typically reveals T2 hyperintensity and restricted diffusion in affected spinal cord segments [5,6]. Localized vasculitis is also a possible cause of neurological complications after TACE, with more delayed symptoms and normal spinal magnetic resonance findings [8]. However, early imaging may be negative within the first 24 hours, even in the case of embolism, necessitating clinical vigilance.

There is no standardized treatment for spinal cord infarction post-TACE. High-dose corticosteroids are commonly administered based on protocols for acute spinal cord injury, although their efficacy remains controversial in non-traumatic cases, mostly due to their adverse effects (e.g., gastrointestinal ulcer, concurrent infection) [9,10]. A Cochrane meta-analysis showed marked improvement in motor and functional status of patients who received high-dose corticosteroid therapy within the first 24 hours and extending to 48 hours after spinal cord injury, even if treatment was not initiated within the first 3 hours, without increasing morbidity and mortality [11]. Despite a lack of randomized studies, clinical evidence from case reports and inference from studies on the treatment of non-traumatic spinal cord injuries have shown that steroid therapy may be beneficial to patients with post-TACE spinal cord injury. Most cases with spinal cord ischemia after TACE in the literature received high-dose steroid therapy with various levels of improvement at the time of report (Table 1) [5,6,8,12]. Two deaths were recorded, one due to HCC rupture, which may not be related to the use of steroids, and the other due to aspiration pneumonia and sepsis [5,13]. To minimize the risk of adverse events from steroid use, careful monitoring and prophylactic measures, such as antibiotics for suspected infection, and proton-pump inhibitors to prevent gastrointestinal complications, should be indicated. Finally, early rehabilitation, including physical therapy and bladder/bowel management, plays a crucial role in functional recovery [5-8].

**Table 1:** Characteristics, treatment, and outcome of patients with post-TACE spinal cord injury in the literature. MRI: magnetic resonance imaging, HCC: hepatocellular carcinoma, NA: not available; TACE: transarterial chemoembolization

Author	Gender/Age	Elapsed Time Until First Symptoms (Hours)	Spinal MRI Findings	Medication	Physical Therapy	Outcome
Praditukrit et al. (2024) [7]	Male/63	Immediately after procedure	Hyperintense lesion at T9-T11 on T2-weighted	Supportive care	Yes	Improved motor function, no improvement in bladder and sensory function
Hieu et al. (2023) [6]	Male/78	Immediately after procedure	Ischemia at T1-T12	High-dose steroid	Yes	Improved sensory function
Chen et al. (2023) [14]	Male/59	Immediately after procedure	Ischemia at T11-L1	Supportive care	Yes	No improvement in motor function
Lee et al. (2022) [5]	Male/58	6 hours after the procedure	Ischemia at T4-L1	High-dose steroid	No	Death after 3 days due to HCC rupture
Bazine et al. (2014) [12]	Female/62	Immediately after procedure	Ischemia at T10	High-dose steroid	Yes	Improved sensory function, no motor function improvement
Tufail et al. (2010) [8]	Male/45	8 hours after procedure	No abnormal finding	High-dose steroid	Yes	Completely recovered
Kim et al. (2010) [13]	Male/65	8 hours after procedure	Some increase in intensity lesion at T9 on T2-weighted	Dexamethasone	NA	Death after 3 months due to pneumonia and sepsis
Kim et al. (2010) [13]	Male/55	6 hours after the procedure	Increase intensity lesion at T9-T10 on T2-weighted	Dexamethasone	NA	Almost recovered after 2 months
Our case (2024)	Male/33	Immediately after procedure	Ischemia at T9	High-dose steroid	Yes	Almost recovered after 8 months

The patient in this case report is the youngest to have been afflicted by this complication, as reported in the literature. This complication is especially debilitating and life-changing for younger patients. Treatment including high-dose corticosteroid infusion and early physical therapy to preserve and restore motor, sensory, bladder and anal function. While there is little evidence for or against the use of corticosteroids, given the rarity of the complication, it would be impossible to have a larger study or clinical trial to determine its effectiveness. Thus, this seems to be a suitable strategy for patients with this complication.

## Conclusions

Although TACE is generally a safe procedure, it carries a possible risk of severe neurological complications, including spinal cord infarction. Clinicians should be aware of potential extrahepatic arterial supply to HCC lesions and consider preventive measures to minimize embolization-related risks. This complication should be included during discussion with patients before the procedure, especially for those who have had previous TACE sessions. High-dose corticosteroids have been used for most cases in the literature, and it was the rationale for their use in this patient. However, it is hard to tell whether steroid treatment contributes to his partial recovery.
